# Editorial: Evolvability, Environments, Embodiment & Emergence in Robotics

**DOI:** 10.3389/frobt.2018.00103

**Published:** 2018-08-31

**Authors:** John H. Long, Eric Aaron, Stéphane Doncieux

**Affiliations:** ^1^Interdisciplinary Robotics Research Laboratory, Vassar College, Poughkeepsie, NY, United States; ^2^Departments of Biology and Cognitive Science, Vassar College, Poughkeepsie, NY, United States; ^3^Department of Computer Science, Vassar College, Poughkeepsie, NY, United States; ^4^Department of Computer Science, Colby College, Waterville, ME, United States; ^5^UMR 7222, ISIR, Sorbonne Universités, UPMC Univ Paris 06, Paris, France

**Keywords:** evolvability, environments, embodiment, emergence, robustness, cognitive robotics, evolutionary robotics

We challenged researchers to grapple with four ideas and their interactions—evolvability, environments, embodiment, and emergence. These are complex drivers underlying the designs and actions of autonomous, mobile, and physical systems. How does a robot or robotic system come to have intelligent, goal-directed behavior? This question is, indeed, a grand challenge.

The articulation of a “grand challenge” frames a field's most important goals and the methods to achieve them. Grand challenges launched the *Frontiers in AI and Robotics* specialty sections of evolutionary robotics (Eiben, 2014), virtual environments (Slater, 2014), and computational intelligence (Prokopenko, 2014). Eiben (2014), for example, suggests three grand challenges: (1) the automatic generation of novel, original robots that are surprising in their design to humans, (2) self-reproduction in physical robots, and (3) open-ended evolution of physical robots in an open-ended environment. Stressing the complex, integrated nature of physical robots operating in the physical world, Doncieux et al. (2015) elaborate a methodological approach, emphasizing experiments designed to test specific hypotheses. This methodology is the hallmark of the work presented in this research topic, with the demands of rigorous experimentation forcing investigators to operationalize ideas.

Robust, expository experiments are founded upon robust, expository models. Integrating the domains of cognition, motion, and time into a hybrid system, Aaron creates the Dynamical Intention-Hybrid Dynamical Cognitive Agent (DI-HDCA) modeling framework. Across a range of task environments, the embodied DI-HDCA agents demonstrate behavior that is emergent, the on-going result of everything from micro-cognitive processes to embodied physical actuation interacting in a physical world. Explicitly modeling both continuous dynamics of and discrete transitions between behaviors allows the investigator to probe how DI-HDCAs continuously search for real-time reactive, goal-directed solutions.

Finding optimal solutions in a search space is also the goal of evolutionary robotics (ER), which enables computational evolution to optimize over a fitness function. In biological evolution, such fitness-guided search generates a large diversity of species across ecological niches; in ER, generating diverse solutions is not always emphasized, but diversity can impact both the evolvability of populations and the robustness of individual agents, promoting exploration of multiple behavior spaces and choice among multiple behaviors. Pugh et al. investigate quality-diversity (QD) algorithms, which aim to discover all possibilities by rewarding the evolution of novelty. With case studies of robot maze navigation, they show how hybridized behavioral characterizations in QD algorithms may be key for advancing evolutionary exploration and, ultimately, evolvability.

While evolvability has many definitions, ranging from current adaptability to future capacity for innovation (Pigliucci, 2008), Lehman et al. argue that ER needs to focus on innovative creation. Creative potential is studied productively as a property of populations since they, not individuals, are the entities that evolve. As collections of related individuals, populations vary, and that variance provides the range of phenotypes upon which selection acts. But directional selection, which optimizes locally, reduces variance, slowing adaptation and evolvability. The authors show that divergent selection can generate variance within a population, increasing variance in the short term. Alternating between directional and divergent selection can mediate between local adaptation and global exploration.

Another potential way to increase evolvability is to find mechanisms that drive behavioral specialization within a population. Montanier et al. take on the challenge, investigating agents that can evolve specialized foraging behaviors for the acquisition of two different resources. Reproductive isolation, however it might be achieved, appears to be key to specialization. Thus, most importantly for ER, mating algorithms and scenarios should be treated carefully and justified, given their potential for creating and maintaining variance within a population. Mating and selection are separate evolutionary drivers.

Evolvability also depends on morphology. Cappelle et al. demonstrate that structural modularity also impacts the efficacy of evolution, when robust behavior is the goal of the search. Comparing modular and non-modular architectures, they found that robots with modular morphologies and controllers can more quickly adapt to new environments. Most promising is the connection of modularity to function: modules shaped by previous evolutionary history are predisposed to detect percepts in new combinations and new environments. Thus, evolved morphology that is modular, if present and preserved, endows a population with greater evolvability, as predicted by the Wankelmut benchmark (Schmickl et al., 2016).

Modularity, however, and evolvability by extension, need not be a direct target of selection. In a population of networks, for example, selection for a combination of performance and reduced connection costs evolves modularity (Clune et al., 2013). Such indirect evolution of modularity is tested for the first time in physical robots by Livingston et al.: They select for enhanced phototaxis of surface-swimmers, in which the genome encoded 60 weights of neural networks connecting photoresistors to motor outputs. With selection on behavior alone, over the course of 10 generations, the primary target of selection is network sparsity; modularity is a correlated evolutionary by-product. This work broadens our understanding of conditions under which modularity may evolve.

Evolution also depends on development. In addition to genetic processes, development introduces epigenetic operators that govern the mapping of genes into morphologies. Using physical robots, Brawer et al. create a developmental process for wiring sensors to motors. In one population, development is altered by wires' physical interactions; in another, those physical interactions are avoided. From identical starting points, these two different epigenetic operators guide different evolutionary responses, changes over generational time that are mediated by the epigenetic process of building working physical robots. This is the first demonstration employing physical robots to show that epigenetic operators can be created and used to complicate, in explicit ways, evolutionary search.

Let us return to our grand challenge: How does a robot or robotic system come to have intelligent, goal-directed behavior? This research topic identifies key processes: (1) the principled emergence of goal-directed behavior from a hierarchy of dynamical processes (Aaron); (2) opportunities for enhanced evolvability and robustness from selection for diversity in populations (Lehman et al.; Pugh et al.); (3) the evolution of specialized behavior from reproductive isolation (Montanier et al.); (4) the evolution of robust behavior from modular systems, which may be evolved indirectly (Capelle et al.; Livingston et al.); and (5) the creation of phenotypic variation and enriched evolutionary possibilities from epigenetic developmental processes (Brawer et al.).

The intersection and interaction of these mechanisms provides ample opportunity to explore the evolution of intelligent behavior. The search continues for principled approaches for their integration in physically embodied systems, biological, or computational.

## Author contributions

All authors listed have made a substantial, direct and intellectual contribution to the work, and approved it for publication.

### Conflict of interest statement

The authors declare that the research was conducted in the absence of any commercial or financial relationships that could be construed as a potential conflict of interest.
